# Supplementary material for: Timeliness of Yellow Fever Specimen Collection and Transport in Ghana, 2018-2022
Source: medRxiv. 2025 Jun 20:2025.06.19.25329877. Preprint. [Version 1] doi: 10.1101/2025.06.19.25329877 (PMC12204298; doi:10.1101/2025.06.19.25329877)
Supplement: Supplement 2 — S1 Fig. Ghana Districts and Regions 2018-2022 The 260 districts and 16 regions in Ghana that were analyzed for yellow fever testing from 2018-2022 are shown with the National Public Health and Reference Laboratory (NPHRL). [file media-2.docx]

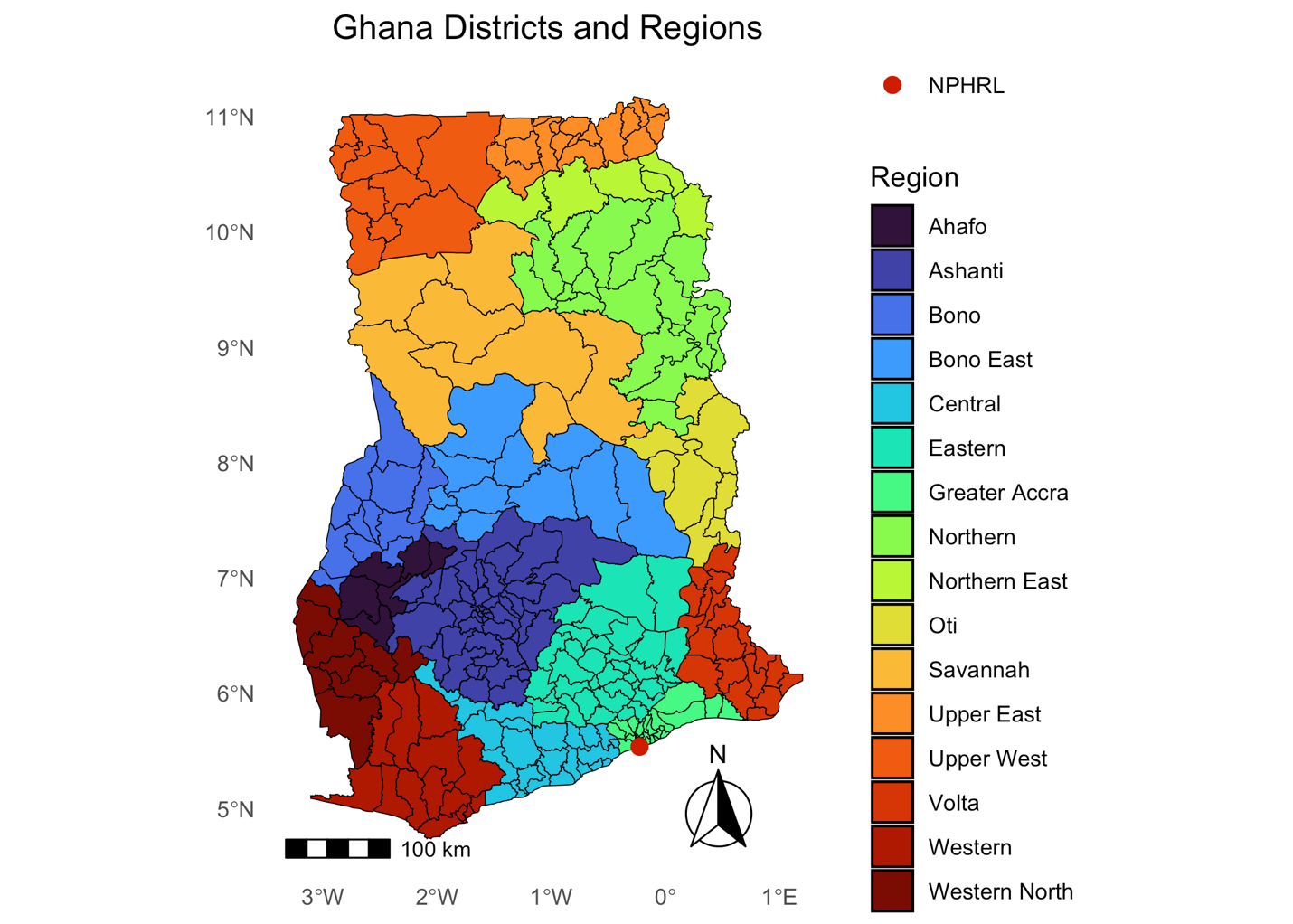


**S1 Fig. Ghana Districts and Regions 2018-2022**

The 260 districts and 16 regions in Ghana that were analyzed for yellow fever testing from 2018-2022 are shown with the National Public Health and Reference Laboratory (NPHRL).
